# LOAD1 and LOAD2: Longitudinal characterization of mouse models carrying human‐relevant risk factors for late‐onset Alzheimer’s disease

**DOI:** 10.1002/alz.091348

**Published:** 2025-01-03

**Authors:** Cristian S Bernabe, Kevin P Kotredes, Ravi S Pandey, Gregory W Carter, Michael Sasner, Adrian L Oblak, Gareth R Howell, Bruce T. Lamb, Paul R Territo, MODEL‐AD consortium

**Affiliations:** ^1^ Indiana University School of Medicine, Indianapolis, IN USA; ^2^ Stark Neurosciences Research Institute, Indianapolis, IN USA; ^3^ The Jackson Laboratory, Bar Harbor, ME USA; ^4^ The Jackson Laboratory for Genomic Medicine, Farmington, CT USA; ^5^ Stark Neurosciences Research Institute, Indiana University School of Medicine, Indianapolis, IN USA

## Abstract

**Background:**

Alzheimer’s disease (AD) is the most common form of dementia, yet the effectiveness of disease‐modifying interventions is inconclusive. Although exceptional progress in our understanding of AD neuropathology has been made via transgenic mouse models bearing familial mutations, they often fail to recapitulate the disease progression of late‐onset AD (LOAD). To address this, MODEL‐AD has developed LOAD1 and LOAD2 mouse models which carry the most common human‐relevant risk factors for AD. In‐depth, longitudinal characterization through aging will reveal useful insights to develop novel treatments for LOAD.

**Method:**

APOEε4 and Trem2*R47H, two risk factors for LOAD, were incorporated into C57BL/6J mice to produce the double homozygous LOAD1 model, whereas LOAD2 also contains humanized amyloid‐beta (Aβ) yielding a triple homozygous model. Cohorts of LOAD1 and LOAD2 mice were aged on multiple sites to 4‐, 12‐, 18‐, and 24‐month timepoints and both sexes were characterized using behavior, PET/CT, cytokines/Aβ40‐42 immunoassays, and astrocyte and microglia immunohistochemistry.

**Result:**

Although aging LOAD1 and LOAD2 mice did not display significant cognitive deficits, there was a genotype‐dependent increase of plasma levels of Ab40‐Ab42. Both sexes across genotypes showed significant region‐dependent increases in brain glycolysis and tissue perfusion between 4 and 24 months. Consistent with the increased brain metabolism and perfusion neurovascular coupling phenotypes, immunopathology analyses revealed an age‐dependent increased number of astrocytes across genotypes that was restricted to cortical regions. Similarly, the total number of activated microglia was slightly elevated in cortical regions of aging mice, even though the results were only marginally significant. Lastly, aged LOAD1 mice displayed increased brain levels of the pro‐inflammatory cytokine IL‐12p70 when compared to LOAD2. Longitudinal analyses of brain and plasma cytokines are still in progress.

**Conclusion:**

LOAD1 and LOAD2 PET/CT analyses revealed phenotypes which are in line with imaging profiles of patients at prodromal stages of AD. Combined with the astrogliosis, these strains are promising venues that can be used to test early disease‐modifying therapeutic targets and can also serve as platform to incorporate additional human‐relevant AD risk factors.

